# An exploratory clinical trial of preoperative non-invasive localization before breast-conserving surgery using augmented reality technology

**DOI:** 10.1007/s10549-024-07272-3

**Published:** 2024-05-14

**Authors:** Minah Lee, Joohyun Woo, Se Hyun Peak, Hyun Goo Kim, Woo Sung Lim, Jin Chung, Jee Eun Lee, Jeoung Hyun Kim, Sanghui Park, Ji Min Kim, Jun Woo Lee

**Affiliations:** 1 Department of Radiology, School of Medicine, Ewha Womans University, Seoul, South Korea; 2 Division of Breast Surgery, Department of Surgery, School of Medicine, Ewha Womans University, Seoul, South Korea; 3 Department of Pathology, School of Medicine, Ewha Womans University, Seoul, South Korea

**Keywords:** Breast cancer, Breast conservative surgery, 3D breast model, Augmented reality

## Abstract

**Purpose:**

This single-center, randomized, prospective, exploratory clinical trial was conducted to assess the clinical efficacy of an augmented reality (AR)—based breast cancer localization imaging solution for patients with breast cancer.

**Methods:**

This clinical trial enrolled 20 women who were diagnosed with invasive breast cancer between the ages of 19 and 80, had a single lesion with a diameter ≥ 5 mm but ≤ 30 mm, had no metastases to other organs, and had not received prior chemotherapy. All patients underwent mammography, ultrasound, computed tomography, and magnetic resonance imaging for preoperative assessment. Patients were randomly assigned to ultrasound-guided skin marking localization (USL) and AR-based localization (ARL) groups (*n *= 10 in each group). Statistical comparisons between USL and ARL groups were made based on demographics, radiologic features, pathological outcomes, and surgical outcomes using chi-square and Student t-tests.

**Results:**

Two surgeons performed breast-conserving surgery on 20 patients. Histopathologic evaluation of all patients confirmed negative margins. Two independent pathologists evaluated the marginal distances, and there were no intergroup differences in the readers' estimates (R1, 6.20 ± 4.37 vs. 5.04 ± 3.47, *P* = 0.519; R2, 5.10 ± 4.31 vs. 4.10 ± 2.38, *P* = 0.970) or the readers' average values (5.65 ± 4.19 vs. 4.57 ± 2.84, *P* = 0.509). In comparing the tumor plane area ratio, there was no statistically significant difference between the two groups in terms of either reader's mean values (R1, 15.90 ± 9.52 vs. 19.38 ± 14.05, *P* = 0.525; R2, 15.32 ± 9.48 vs. 20.83 ± 12.85, *P* = 0.290) or the overall mean values of two readers combined (15.56 ± 9.11 vs. 20.09 ± 13.38, *P* = 0.388). Convenience, safety, satisfaction, and reusability were all superior in the AR localization group (*P* < 0.001) based on the two surgeons' responses.

**Conclusion:**

AR localization is an acceptable alternative to ultrasound-guided skin marking with no significant differences in surgical outcomes.

**Supplementary Information:**

The online version contains supplementary material available at 10.1007/s10549-024-07272-3.

## Introduction

As breast-conserving surgery (BCS) has become commonplace, localization has become essential during surgery for non-palpable breast cancer [1]. With the recent widespread adoption of health checkups, the rate of detection of low-stage, non-palpable breast cancer is increasing [2, 3]. In BCS, it is crucial to employ a technique that allows for precise resection of the targeted surgical area while minimizing the extent of tissue removal [4, 5]. The tumor’s location is accurately identified to minimize the scope of surgical intervention, and the procedure is carried out with a carefully calculated margin around the tumor. Localization is now part of the process, and a radiologist can perform wire localization or ultrasound (US)–guided skin marking. Wire localization is a method of inserting a wire into the center of a lesion under US or mammography guidance. However, wire localization may cause additional pain in preoperatively anxious patients. Furthermore, wires can become dislodged, shifted, or fractured. Additionally, cosmetic problems may occur due to non-optimal incision arrangement, depending on the position of the wire [6–8].

US-guided skin marking is a technique that employs US imaging to pinpoint the tumor’s location and mark it on the patient’s skin. As a non-invasive approach, this method offers the benefit of causing no pain to the patient, making it a popular choice for preoperative localization. However, one drawback is that the marked location on the skin and the actual tumor position may shift depending on the patient’s posture, leading to potential inaccuracies.

Augmented reality (AR) is a technology that overlays virtual objects onto the real world after spatial registration. Facilitating the enhancement of real-world environments, AR offers the potential to be integrated into the breast localization process [9]. Recent studies have shown that visualization of breast lesions using AR can be helpful in breast localization [10]. SKIA-Breast (SKIA Inc., Seoul, South Korea) uses AR technology to create three-dimensional (3D) models based on breast cancer information shown on chest computed tomography (CT). SKIA-Breast is designed so that the operating surgeon can confirm the lesion’s exact location using information that combines the 3D model and the actual patient’s bodily information in the operating room. Since many people with breast cancer in Korea undergo chest CT for preoperative staging, no additional examination is required to create a 3D model. Additionally, when chest CT is performed, the patient’s posture is similar to the supine position typically required in the operating room, allowing for immediate use of the CT images for modeling purposes.

This study, as an exploratory clinical study of SKIA-Breast, evaluated the effectiveness of the SKIA-Breast surgical guidance system as a non-invasive breast tumor localization tool, in comparison with the US-guided skin marking process.

## Methods

Ethical approval was obtained from our institutional review board (IRB No. 2021-09-020), and patients provided informed consent for the use of their clinical and imaging data, including randomization into either the study (using ARL) or control group.

### Study sample

In a prospective clinical trial conducted at our institution from March 2022 to March 2023, we selected patients diagnosed with invasive breast cancer who were scheduled for breast cancer surgery and were between 19 and 80 years old. Additionally, mammography, US, chest CT, and magnetic resonance imaging (MRI) were all performed for preoperative evaluation, and single lesions between 5 and 30 mm in diameter defined eligibility for participant inclusion. Patients were excluded if the cancer had metastasized to other organs or if they had undergone prior chemotherapy.

**Fig. 1:**
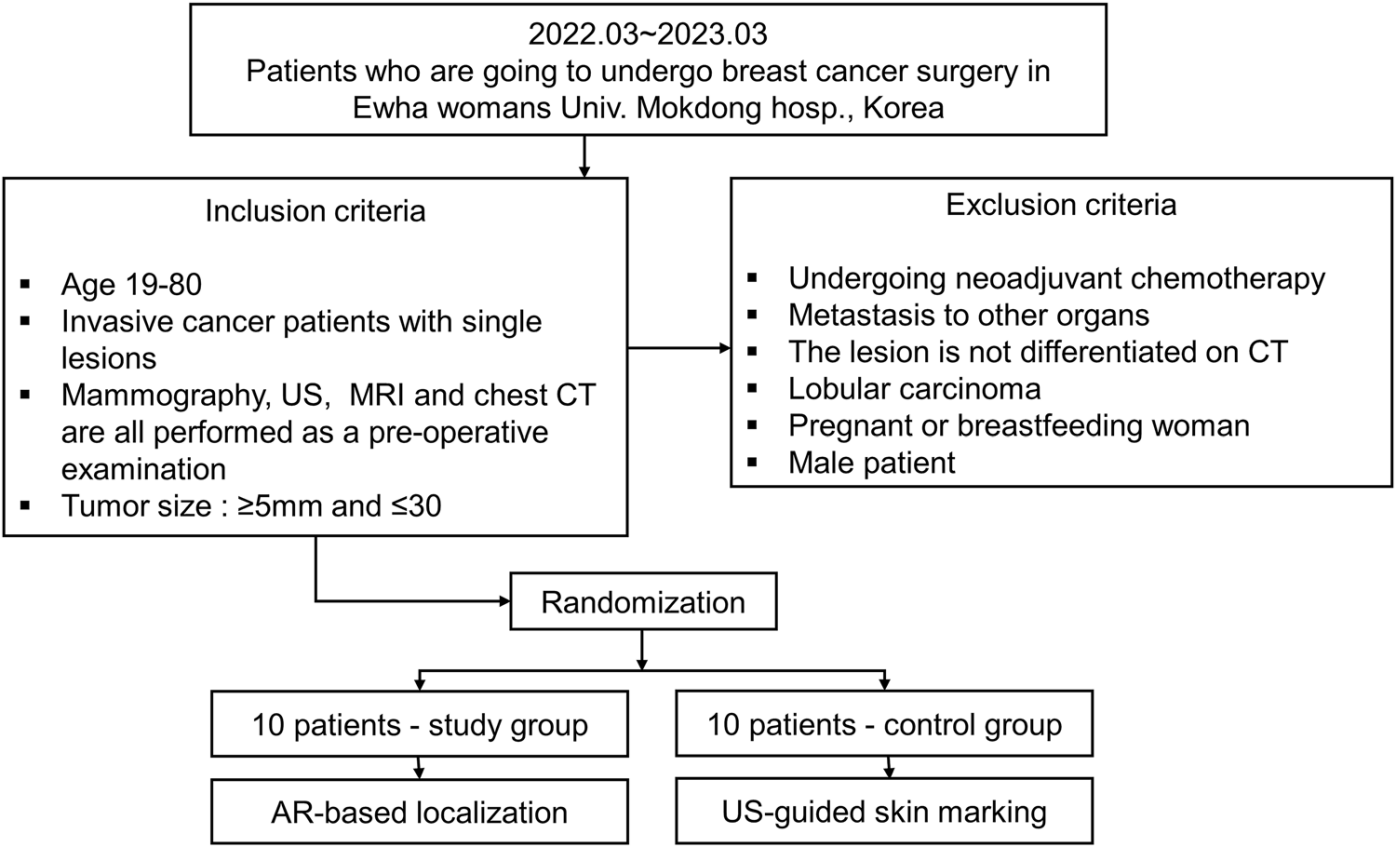
Flow chart of the study population

Exclusion criteria included patients with contraindications for MRI or CT scans, those who displayed no lesions in CT imaging, pregnant or breastfeeding individuals, biologically male patients, those who had previously undergone chemotherapy or had metastases to other organs, and individuals with pathological biopsy results indicating invasive lobular carcinoma. Eligible patients were randomized within 48 h prior to surgery using a block randomization approach guided by a random number table. Patients were allocated to either the study or control group at a 1:1 ratio with patient consent, with ten patients assigned to each group (Fig. 1).

### Imaging evaluation

For preoperative evaluation, mammography, US, MRI, and chest CT were performed. Digital mammography (Dimension, Hologic, Bedford, MA, USA) was performed on both breasts, including craniocaudal and mediolateral oblique views. Breast US was performed using a 7.5–15-MHz linear-array transducer with an iU22 scanner (Philips Medical Systems, Bothell, WA, USA) and the Aixplorer system (Supersonic Imagine, Aix en Provence, France). MRI was performed using a 3.0-Tesla MR system (Magnetom Vida, Siemens Medical Solutions, Forchheim, Germany) using a dedicated 128-channel breast coil. The MRI protocol was a full protocol comprising a T2-weighted sequence, one pre-contrast T1-weighted sequence, and six post-contrast T1-weighted sequences. Diffusion-weighted images (b-value of 0, 800) and kinetics analysis were performed.

Chest CT scans for breast cancer patients before surgery are covered by national health insurance in Korea. Therefore, in our institution, surgeons perform chest CT scans on many breast cancer patients undergoing preoperative evaluations, considering the assumed clinical stage, symptoms, smoking history, medical history, and family history. All CT scans were obtained with the patient in the supine position and using either of two 64-channel CT scanners (Sensation 64; Siemens Medical Solutions, Forchheim, Germany) (SOMATOM Definition Flash; Siemens Medical Solutions, Forchheim, Germany). The CT scans were obtained 40 s after intravenous injection of 100 mL of a non-ionic contrast agent (iohexol, Bonorex 350; CMS, Seoul, Korea) at a rate of 2.3 mL/s with power injection.

### AR-based localization using SKIA-breast

**Fig. 2:**
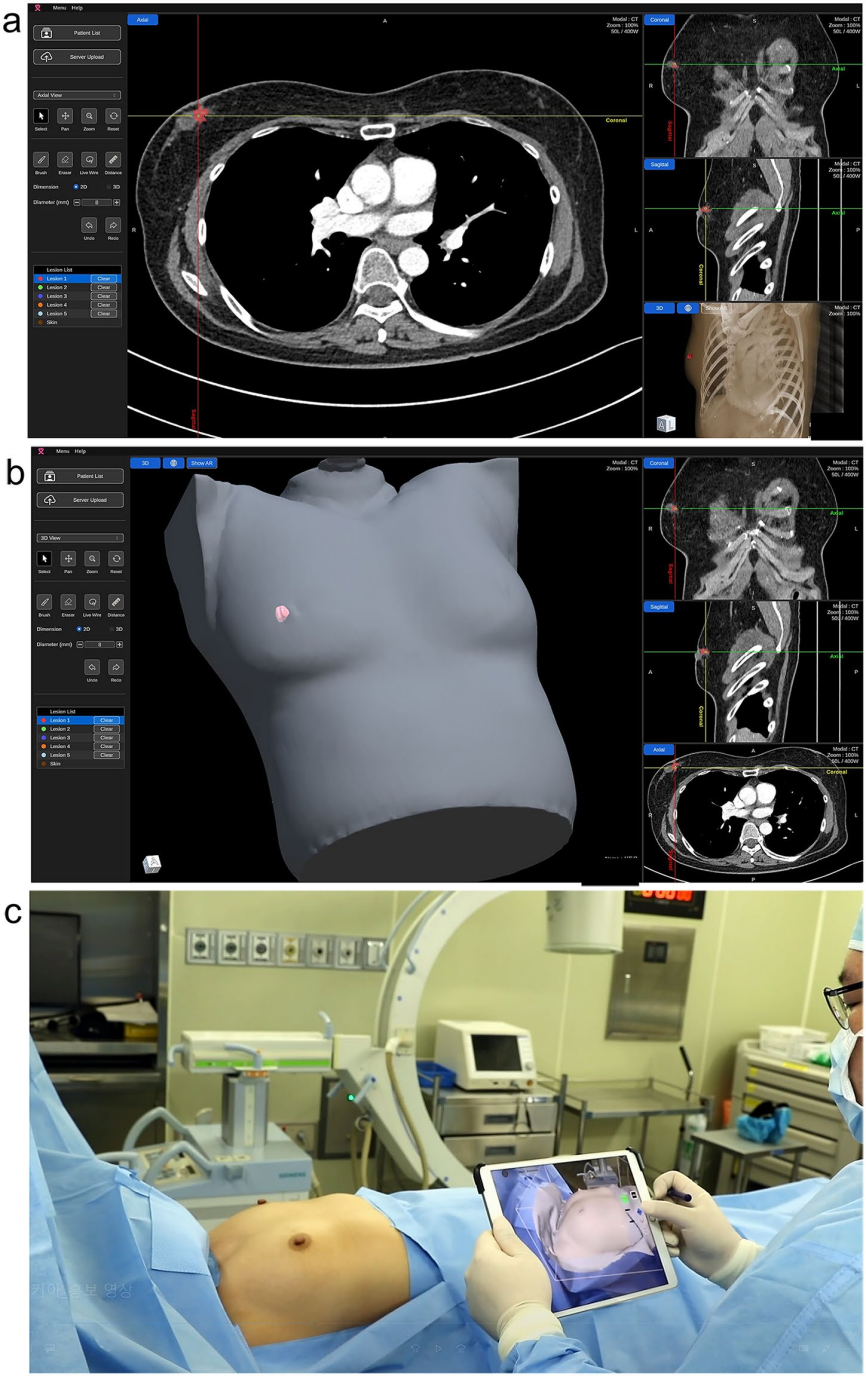

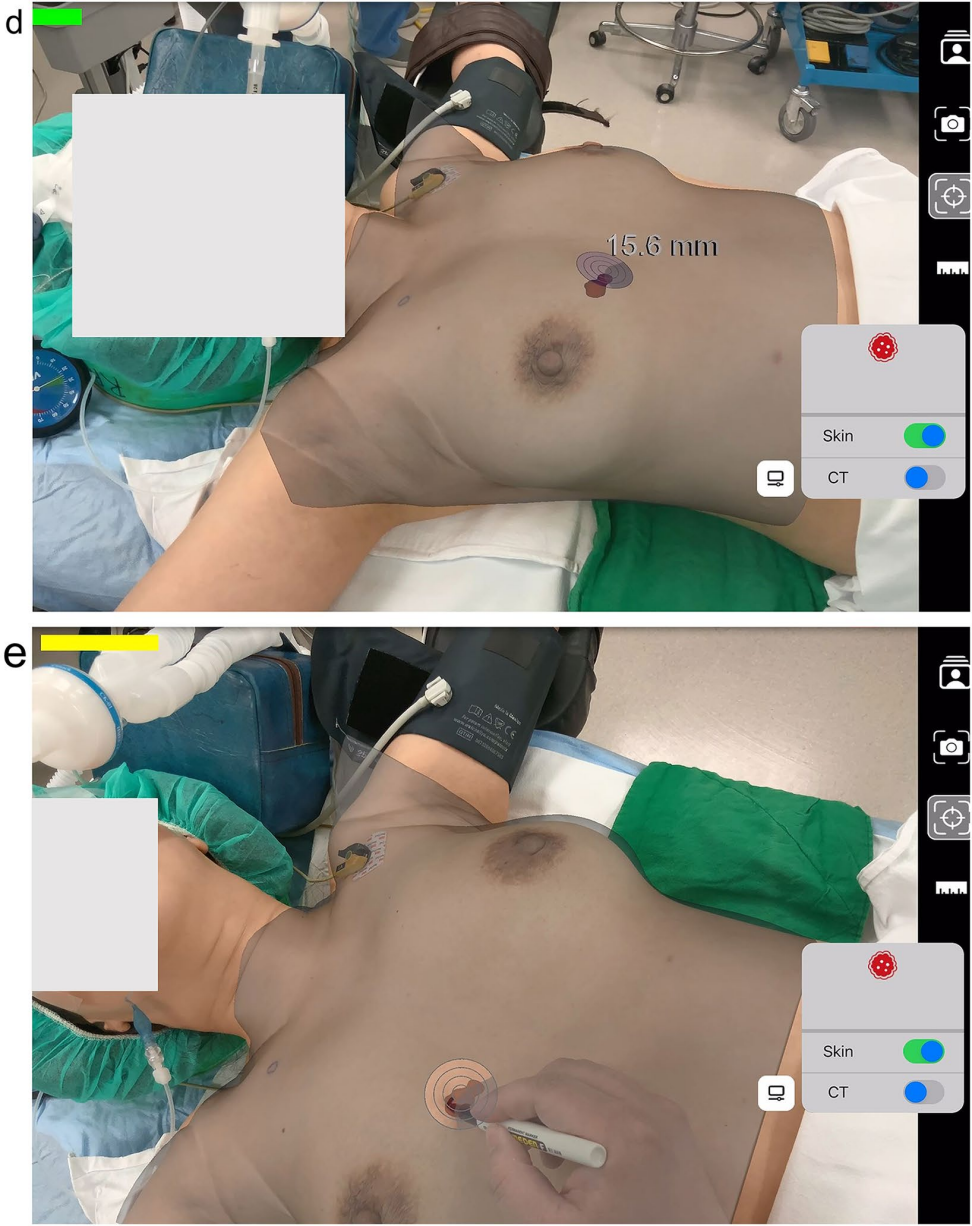
Steps of AR-based localization using SKIA-Breast. **a** Segmentation of the anatomical structure is performed by drawing the border of the breast tumor identified on the chest CT image using the SKIA processor. **b** An AR 3D model is automatically created by the SKIA processor. **c** On the day of surgery, after preparations are complete, the surgeon uses the 3D camera mounted on the iPad and the SKIA application to position the patient’s upper body within the rectangular parallelepiped displayed on the screen. **d, e** capture images from iPads running the SKIA application. After generating data that matches the 3D models created by the SKIA processor and SKIA application, you can use a 3D camera to view the virtual locations of the patient’s body and breast tumors on the screen; depth information is also displayed numerically. The surgeon checks the position on the screen and marks the boundary with a pen directly above the position indicated by the tumor

The application of SKIA-Breast requires the patient to undergo preoperative contrast-enhanced chest CT. In this study, three board-certified breast radiologists (J.C, L.J.E, K.J.H; 14–16 years of experience) matched the location of breast tumors on chest CT and used the SKIA processor, a web-based software application, to perform segmentation and delineate the breast tumor margins identified using chest CT images. 3D AR models were automatically created by the SKIA processor and uploaded to the SKIA server. On the day of surgery, the patients underwent their respective procedures in the supine position, similar to the posture taken for capturing the preoperative CT scans. After the completion of preoperative preparations, such as disinfection, the surgeon used a 3D camera (mounted on an iPad [Apple, Cupertino, CA, USA]) and the SKIA application to align the patient’s upper body inside a rectangular parallelepiped on the screen. Once the 3D model generated by the SKIA processor was matched with the 3D model created within the SKIA application, the 3D camera was used to project light onto the patient’s body. This allowed the virtual position of the breast tumor, along with the patient’s body, to be displayed on the screen. At that time, depth information was presented numerically on the display. The surgeon then verified the location and used a pen to mark the skin directly above the targeted area. BCS was carried out based on these markings (Fig. 2) (Supplementary Information).

### US-guided skin marking localization

For the patients assigned to the control (US-guided skin marking localization, USL) group, the radiologist performed US-guided skin marking on the breast lesions on the day before or the morning of surgery. Three radiologists with 14–16 years of experience first identified the location of the breast tumor using US (iU22). After the lesion’s position was confirmed, its margins were traced with a pen used to mark the directly overlying skin. These markings were used to guide the BCS.

### BCS and resection margin evaluation

SKIA-Breast was applied to 10 patients assigned to the study (AR-based localization, ARL) group, and US-guided skin marking was applied to 10 patients assigned to the control (USL) group before surgery. Two experienced surgeons (J.W.L. and W.S.L) with 11 and 20 years of experience performed the BCS. After BCS, two pathologists (P.S.H. and K.J.M., blinded to the group assignments and other study data) with 5 and 16 years of experience independently evaluated the specimens’ reservation margin distance, gross area, and tumor area. Based on this, the ratio of tumor area to gross plane area (formula: tumor plane area/gross (tumor + margin) plane area × 100; plane area calculation followed the elliptical calculation method (primary axis diameter × minor axis diameter × π/4)) was determined.

Given that it was not feasible to blind the surgeons performing the operations to the group assignments, three independent evaluators (P.S.H., W.J.H., K.H.G., each with 6–11 years of surgical experience) were assigned to assess the need for reoperation. The evaluators were blinded to participant information, including group assignments. If the first and second evaluators disagreed, the opinion of the third evaluator was considered decisive.

For the two surgeons who carried out the procedures, a satisfaction questionnaire was administered to evaluate the preoperative breast tumor localization methods. Aspects such as convenience, safety, satisfaction, and potential for reusability were compared between the two groups. A 5-point Likert scale was employed to measure satisfaction; higher scores indicated greater satisfaction. The scale was as follows: 1 point for “fully disagree,” 2 points for “disagree,” 3 points for ‘neither agree nor disagree,” 4 points for “agree,” and 5 points for “fully agree.”

### Statistical methods

The USL and ARL groups were compared based on demographics, clinical factors, and surgical outcomes using chi-square and Student t-tests. Statistical analysis was performed using SPSS Statistics for Windows, version 24.0 (IBM Corp., Armonk, NY, USA). *P*-values < 0.5 were considered statistically significant.

## Results

There were no significant differences between the ARL and USL groups in terms of various demographic and clinical characteristics, such as age, height, weight, menopausal status, family history of breast cancer, palpable symptom at the time of breast cancer detection, and histopathologic features (clinical stage, T stage, nodal status, hormone receptor [ER, PR] status, Her-2 status, KI-67 status, and histologic grade) (Table 1).

**Table 1:** Demographics and imaging features

Variable		Control group (*N* = 10)	Study group (*N* = 10)	P value^(a)^
Age		54.10 ± 9.34	54.50 ± 8.63	0.6215
Median		51.00 (47.00, 62.00)	54.00 (48.00, 61.00)	
Height		157.23 ± 8.75	158.91 ± 6.10	0.739
Median		161.65 (154.70, 164.00)	158.90 (156.10, 161.90)	
Weight		63.04 ± 8.45	60.72 ± 8.30	0.529
Median		62.00 (57.00, 69.70)	60.15 (55.70, 63.00)	
Menopausal status				0.160
Premenopausal		5 (0.5)	2 (0.2)	
Postmenopausal		5 (0.5)	8 (0.8)	
Family history of breast cancer		2 (0.2)	1 (0.1)	0.531
Symptom (palpability)		2 (0.2)	4 (0.4)	0.329
Clinical stage				0.517
IA		7 (0.7)	6 (0.6)	
IIA		3 (0.3)	2 (0.2)	
IIB		0	1 (0.1)	
IIIC		0	1 (0.1)	
T stage				0.606
T1		8 (0.8)	7(0.7)	
T2		2 (0.2)	3(0.3)	
N stage				0.317
N0		10 (1.0)	7 (0.7)	
N1		0	1 (0.1)	
N2		0	1 (0.1)	
Not reported		0	1 (0.1)	
Hormone receptor status				
ER positive		9 (0.9)	9 (0.9)	1.000
PR positive		9 (0.9)	9 (0.9)	1.000
HER 2 positive		1 (0.1)	1 (0.1)	1.000
KI-67		15.20 ± 9.14	19.05 ± 12.45	0.529
Histologic Grade				0.815
Grade 1		1 (0.1)	2 (0.2)	
Grade 2		7 (0.7)	6 (0.6)	
Grade 3		2 (0.2)	2 (0.2)	
Mammography density				0.16
A, B		2 (0.2)	5 (0.5)	
C, D		8 (0.8)	5 (0.5)	
cancer size on US (mm)		15.20 ± 5.16	14.60 ± 5.66	0.684
Invasive size (mm)		16.60 ± 5.74	17.18 ± 11.58	0.796

(a) The mann-Whitney test was used for P value calculation for age, height, weight, KI-67, cancer size on US and invasive size. Chi-square test was used for other categorical variables(Menopausal status, Family history of breast cancer, Symptom, Clinical stage, N stage, Hormone receptor status, Histologic Grade, Mammography density)

*US * ultrasonography

Regarding immunohistological characteristics, among the 20 patients who participated in the study, 16 were categorized as having luminal A subtype, two had luminal B, and two were triple-negative. Radiologically, 19 of the patients displayed a mass indicative of cancer, and one patient presented with a non-mass lesion with no discernible enhancement on chest CT scans. In this patient, there was no problem in identifying the boundaries of the lesion by referring to other modalities, such as US and MRI, so that they could be included in the study. This particular patient was allocated to the USL group during the randomization process (Table 2).

**Table 2:** Characteristics including imaging findings of study patients

Group	No.	Age	Breast density	Symptoms(palpability	ER	PR	Her-2	Clinical stage	MG		US		MRI		CT
									MG finding	calcification	US finding	vascularity	MRI findings	Kinetics	
ARL	1	47	C	(+)	(+)	(+)	(−)	2B	Mass	Absent	Mass	Present	Irregular spiculated enhancing mass	Rapid washout	Enhancing mass
	2	55	B	(−)	(+)	(+)	(−)	1 A	Mass	Absent	Mass	Present	Irregular spiculated enhancing mass	Rapid washout	Enhancing mass
	3	61	B	(+)	(−)	(−)	(−)	1 A	Mass	Absent	Mass	Present	Irregular enhancing mass	Rapid washout	Enhancing mass
	4	48	C	(−)	(+)	(+)	(−)	2 A	Mass	Absent	Mass	Present	Irregular enhancing mass	Rapid washout	Enhancing mass
	5	66	B	(−)	(+)	(+)	(−)	2 A	Mass	Absent	Mass	Present	Irregular circumscribed enhancing mass	Rapid washout	Enhancing mass
	6	53	C	(−)	(+)	(+)	(−)	1 A	Focal asymmetry	Absent	Mass	Present	Irregular enhancing mass	Rapid washout	Enhancing mass
	7	38	D	(−)	(+)	(+)	(+)	1 A	Asymmetry	Absent	Mass	Present	Irregular enhancing mass	Rapid washout	Enhancing mass
	8	60	B	(+)	(+)	(+)	(−)	1 A	Mass	Absent	Mass	Present	Irregular enhancing mass	Rapid plateau	Enhancing mass
	9	53	C	(+)	(+)	(+)	(−)	3 C	Focal asymmetry	Absent	Mass	Absent	Irregular enhancing mass	Rapid plateau	Enhancing mass
	10	64	B	(−)	(+)	(+)	(−)	1 A	Mass	Absent	Mass	Absent	Irregular enhancing mass	Rapid plateau	Enhancing mass
USL	1	46	D	(−)	(+)	(+)	(−)	2 A	Architectural distortion	Absent	Nonmass	Absent	Regional nonmass enhancement	Slow persistent	No enhancement of nonmass
	2	48	D	(−)	(+)	(+)	(−)	1 A	Mass	Absent	Mass	Present	Irregular enhancing mass	Rapid washout	Enhancing mass
	3	63	B	(−)	(+)	(+)	(−)	1 A	Focal asymmetry	Absent	Mass	Present	Irregular enhancing mass	Rapid washout	Enhancing mass
	4	74	B	(+)	(−)	(-)	(−)	2 A	Mass	Absent	Mass	Present	Irregular enhancing mass	Rapid washout	Enhancing mass
	5	54	**C**	(−)	(+)	(+)	(−)	1 A	Mass	Absent	Mass	Present	Irregular enhancing mass	Rapid washout	Enhancing mass
	6	48	C	(−)	(+)	(+)	(−)	2 A	Mass	Absent	Mass	Present	Irregular enhancing mass	Rapid washout	Enhancing mass
	7	49	C	(+)	(+)	(+)	(−)	1 A	Mass	Absent	Mass	Absent	Irregular spiculated enhancing mass	Rapid plateau	Enhancing mass
	8	53	D	(−)	(+)	(+)	(−)	1 A	Focal asymmetry	Absent	Mass	Absent	Irregular enhancing mass	Rapid washout	Enhancing mass
	9	62	C	(−)	(+)	(+)	(−)	1 A	Mass	Absent	Mass	Present	Irregular enhancing mass	Rapid washout	Enhancing mass
	10	46	C	(−)	(+)	(+)	(+)	1 A	Mass	Absent	Mass	Absent	Irregular enhancing mass	Rapid plateau	Enhancing mass

**Fig. 3:**
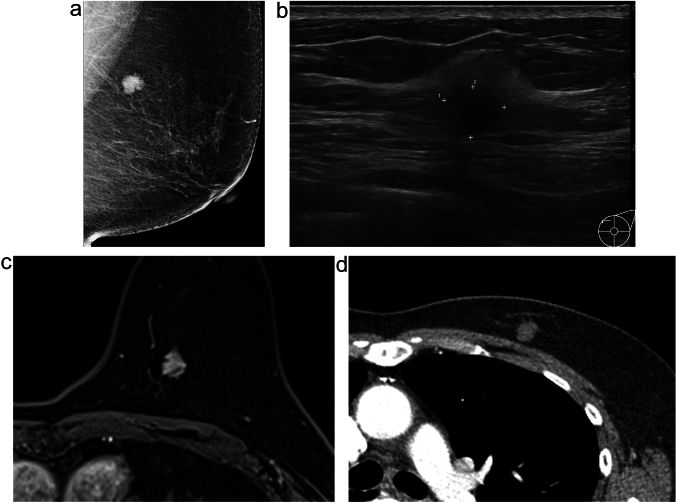
A 60-year-old woman with invasive ductal carcinoma, clinical stage 1 A (ARL No.8). **a** Mediolateral oblique view mammography shows an irregular hyperdense mass in the posterior aspect of the left breast. **b** US shows a 0.8-cm hypoechoic mass in the retromammary fat layer of the left breast. **c** T1-weighted contrast-enhanced, fat-suppressed MRI displays an irregular enhancing mass in the posterior aspect of the left breast. **d** An axial view of contrast-enhanced chest CT shows an enhancing mass in the posterior aspect of the left breast. ARL was applied to the lesion, and the lesion was ultimately diagnosed as invasive breast carcinoma after surgery

**Fig. 4:**
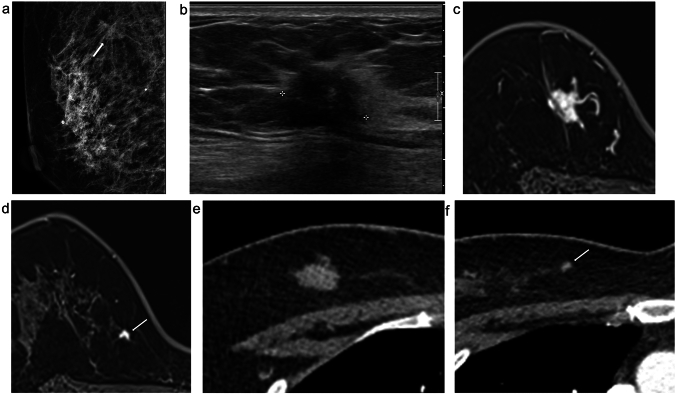
A 48-year-old woman with invasive ductal carcinoma, clinical stage 2 A (ARL No.4); an additional suspicious lesion not seen on US was confirmed on CT, and ARL was applied. **a** Mediolateral oblique view mammography reveals an irregular, spiculated, isodense mass (arrow) in the upper posterior aspect of the right breast. **b** US shows a 1.7 cm irregular hypoechoic mass in the right breast. **c, d** MRI displays an irregular enhancing mass confirmed to be malignant. An additional enhancing mass (arrow) measuring 0.5 cm is visible in the lower inner aspect of the confirmed malignant lesion. A second-look US was performed on this lesion, but it could not be differentiated by US. **e, f** Both the confirmed malignant lesion and an additional suspicious mass (arrow) are visible on CT. ARL was applied to both lesions, and surgery was conducted; both lesions were ultimately diagnosed as invasive breast carcinoma

During the ARL study, two cases demonstrated the effectiveness of preoperative CT scans in lesion localization. Patient 8, who underwent ARL, the tumor was located in the retromammary fat layer, a location posing challenges for conventional wire-guided localization and ultrasound depth perception. Contrast-enhanced CT imaging, as exemplified in Fig. 3, proved invaluable in this scenario by providing superior clarity. Patient 4, who also underwent ARL, a second-look ultrasound was performed to investigate additional suspicious lesions initially observed on MRI. Notably, a previously undetectable lesion on ultrasound was clearly visualized on chest CT scans. Consequently, this additional suspicious mass and the primary lesion were segmented for ARL treatment. Both lesions were later histologically confirmed as invasive carcinoma, as shown in Fig. 4.

Upon independent evaluation of tumor margins by two pathologists, no statistically significant differences were observed between the two groups in terms of margin distance. For the first pathologist, the margin distances were 6.20 ± 4.37 in the USL group vs. 5.04 ± 3.47 in the ARL group (*P* = 0.519). For the second pathologist, the corresponding values were 5.10 ± 4.31 vs. 4.10 ± 2.38, respectively (*P* = 0.970). There was no difference between the two groups in the mean values of the two readers (5.65 ± 4.19 vs. 4.57 ± 2. 84, *P* = 0.509) (Table 3).

**Table 3:** Comparison of margin values evaluated with histopathology results

Margin distance		Control group (*N* = 10)	Study group (*N* = 10)	Total (*N* = 20)	P value
Reader1	Mean ± SD	6.20 ± 4.37	5.04 ± 3.47	5.62 ± 3.88	0.5189^(a)^
	Median	5.50(2.00, 9.00)	5.00(2.00, 7.00)	5.00(2.00, 8.50)	
	At on ink	0 (0.00)	0 (0.00)	0 (0.00)	0.8636^(b)^
	< 2 mm from ink	1(0.1)	2 (0.2)	3 (0.15)	
	2 mm ≤ and < 5 mm	4 (0.4)	2 (0.2)	6 (0.3)	
	5 mm ≤ and < 10 mm	3 (0.3)	4 (0.4)	7 (0.35)	
	10 mm ≤ and < 20 mm	2 (0.2)	2 (0.2)	4 (0.2)	
	20 mm ≤ and < 30 mm	0 (0)	0 (0)	0 (0)	
	30 mm≤	0 (0)	0 (0)	0 (0)	
Reader2	Mean ± SD	5.10 ± 4.31	4.10 ± 2.38	4.60 ± 3.42	0.9695^(a)^
	Median	4.00(3.00, 5.00)	5.00(2.00, 6.00)	4.50(2.00, 5.50)	
	At on ink	0 (0)	0 (0)	0 (0)	0.2862^(b)^
	< 2 mm from ink	2 (0.2)	4 (0.4)	6 (0.3)	
	2 mm ≤ and < 5 mm	6 (0.6)	3 (0.3)	9 (0.45)	
	5 mm ≤ and < 10 mm	1 (0.1)	3 (0.3)	4 (0.2)	
	10 mm ≤ and < 20 mm	1 (0.1)	0 (0)	1 (0.5)	
	20 mm ≤ and < 30 mm	0 (0)	0 (0)	0 (0)	
	30 mm≤	0 (0)	0 (0)	0 (0)	
Reader1 + 2	Mean ± SD	5.65 ± 4.19	4.57 ± 2.84	5.11 ± 3.53	0.5085^(c)^
	Median	5.25(2.50, 6.50)	5.00(2.00, 7.00)	5.00(2.25, 6.75)	
	At on ink	0 (0)	0 (0)	0 (0)	0.6181^(b)^
	< 2 mm from ink	1 (0.1)	2 (0.2)	3 (0.15)	
	2 mm ≤ and < 5 mm	3 (0.3)	2 (0.2)	5 (0.25)	
	5 mm ≤ and < 10 mm	4 (0.4)	6 (0.6)	10 (0.50)	
	10 mm ≤ and < 20 mm	2 (0.2)	0 (0)	2 (0.1)	
	20 mm ≤ and < 30 mm	0 (0)	0 (0)	0 (0)	
	30 mm≤	0 (0)	0 (0)	0 (0)	

(a) Independent two-sample t-test was used for continuous variable

(b) Fisher’s exact test was used for categorical variables was used

(c) Wilcoxon rank-sum test was used

In all the cases examined, no patient was found to have positive margins. Upon review by three independent surgeons, it was concluded that reoperation was unnecessary for any of the patients.

Regarding the mean ratio of the tumor plane area to the gross plane area, there was no statistically significant difference between the two groups according to evaluations by the first reader (15.90 ± 9.52 vs. 19.38 ± 14.05, *P* = 0.525), the second reader (15.32 ± 9.48 vs. 20.83 ± 12.85, *P* = 0.290), or the mean values from both readers (15.56 ± 9.11 vs. 20.09 ± 13.38, *P* = 0.388) (Table 4).

**Table 4:** Comparison of tumor plane area ratio (excluding thickness) to gross plane area among resected tissue sizes

Tumor palne area/gross plane area		Controll group (*N* = 10)	Study group (*N* = 10)	Total (*N* = 20)	P value^(a)^
Reader1	Mean ± SD	15.90 ± 9.52	19.38 ± 14.05	17.64 ± 11.81	0.5247
	Median	15.19	16.67	16.12	
Reader2	Mean ± SD	15.32 ± 9.48	20.83 ± 12.85	18.07 ± 11.35	0.2895
	Median	15.05	19.68	16.39	
Reader 1 + 2	Mean ± SD	15.56 ± 9.11	20.09 ± 13.38	17.82 ± 11.38	0.3875
	Median	16.95	18.40	17.80	

(a) Independent two-sample t-test was used

In the results of the tester satisfaction survey, the ARL group had significantly higher scores than the USL group in terms of convenience, safety, satisfaction, and reusability (*P* = < 0.001). Furthermore, the ARL group’s total scores were statistically higher (*P* = < 0.001) (Table 5).

**Table 5:** Comparison of convenience, safety, satisfaction and reusability of surgeons after applying SKIA-breast and BCS

		Controll group (*N* = 10)	Study group (*N* = 10)	Total (*N* = 20)	P value^(a)^
convenience	Mean ± SD	3.00 ± 0.67	4.60 ± 0.52	3.80 ± 1.01	< 0.001
	Median	3.00(3.00, 3.00)	5.00 (4.00, 5.00)	4.00(3.00, 5.00)	
safety	Mean ± SD	3.50 ± 0.53	4.90 ± 0.32	4.20 ± 0.83	< 0.001
	Median	3.50(3.00, 4.00)	5.00(5.00, 5.00)	4.00(3.50, 5.00)	
satisfaction	Mean ± SD	3.20 ± 0.79	4.70 ± 0.48	3.95 ± 1.00	< 0.001
	Median	3.00(3.00, 4.00)	5.00(4.00, 5.00)	4.00(3.00, 5.00)	
reusability	Mean ± SD	3.30 ± 0.48	4.50 ± 0.53	3.90 ± 0.79	< 0.001
	Median	3.00(3.00, 4.00)	4.50(4.00, 5.00)	4.00(3.00, 4.50)	
Total	Mean ± SD	13.00 ± 2.16	18.70 ± 1.34	15.85 ± 3.41	< 0.001
	Median	13.00(12.00, 15.00)	19.00(17.00, 20.00)	16.50(13.00, 19.00)	

(a) Wilcoxon rank-sum test was used

## Discussion

Localization of non-palpable breast lesions before BCS has increasingly become a critical technique for surgeons aiming for precise and expedient procedures. Existing standard methods for localization include wire-guided, US-guided skin marking, radioactive seed localization, radio-occult lesion localization, magnetic seeds, and carbon tattooing [11]. However, none of these techniques have definitively proven superior in reducing the rate of positive tumor margins. Most are invasive in nature.

Our study employed ARL, a non-invasive technique that enables surgeons to visually confirm the surgical area in the operating room in real time. This method was compared with USL, another non-invasive approach. The study found no significant difference between ARL and USL in terms of margin distance to tumor area ratio on the resected surface. Given its equivalent accuracy and enhanced convenience, ARL offers a valuable alternative to USL for primary users, namely surgeons.

ARL’s distinct advantage lies in its use of preoperative CT scans for localization, making it particularly effective in certain cases. For example, in Patient 8, who underwent ARL, the tumor was located in the retromammary fat layer, a location challenging for wire-guided techniques and depth perception in US. ARL excelled in this context due to the clarity provided by contrast-enhanced CT imaging. The process of AR segmentation using CT scan was simple, and it was a case in which the surgeon could obtain depth information directly by performing ARL (Fig. 3).

In the case of Patient No. 4, ARL was used. A second-look US was also performed to further examine an additional suspicious lesion initially observed on MRI. This particular lesion, which we had not detected, was more clearly identified using chest CT scans, and histologically confirmed as additional invasive ductal carcinoma, postoperatively (Fig. 4). It is worth noting that lesions surrounded by fat or situated at the periphery may not be easily distinguishable using US alone. When multiple lesions require surgical intervention, the detection process can become complex using US scans alone. In such cases, the ARL method, which uses information from chest CT scans, may offer an easier and potentially more accurate localization approach than US-guided methods.

Numerous previous studies have explored the domain of AR-based localization; however, our research marks the first clinical trial that employs a marker-less AR-based approach for localization. In a preliminary study, Gouveia and colleagues performed AR-based localization on a single patient using an OST (optical see-through) AR headset [12]. Their study validated the effectiveness of this non-invasive localization technique, confirming that it achieves an overlapping effect similar to that of carbon tattooing. In contrast, the AR display method used in our research is based on a VST (video-see-through) system using an iPad. iPads are more cost-effective than commercially available optical combiners and offer the benefit of immediate accessibility, as the device is readily available for use. Additionally, in multiple papers by Gouveia et al., the tumor location was verified using MRI scans, and 3D modeling was carried out. Notably, the orientation of the tumor can differ between the prone position taken during a standard breast MRI and the supine position of the patient in the operating room [13–15]. As a result, MRI scans in these studies needed to be captured in the supine position. In the Korean context, chest CT scans are typically conducted in the supine position to confirm distant metastases prior to surgery. Therefore, our study did not necessitate additional imaging, as these existing chest CT scans sufficed for 3D modeling purposes.

Duraes et al. conducted research using an AR display based on the VST method, similarly employing an iPad like in our study [13]. While their investigation compared radioisotopic localization in nine patients and reported successful tumor localization in each breast quadrant, it did not offer a comparison of the actual surgical outcomes. In contrast, our study undertook a more direct comparison by evaluating resection margins for both ARL and USL. We found no significant difference between the two techniques in terms of surgical performance.

When assessing specimen margins across various localization methods in previous studies, the reported accuracy was as follows: carbon marking at 81.1% [16], wire-guided ranged from 70.8 to 87.4% [17–19], ROLL (radio-occult lesion localization) 75–93.5% [17–19], and clip marker 90–92% [20, 21]. Notably, the accuracy for US-guided skin localization ranged from 89 to 97% [21, 22]. In our study, both groups achieved a 100% accuracy rate. For the USL group, this high accuracy can be attributed to the involvement of an experienced radiologist and effective communication between the radiologist and the surgeons. In the case of ARL, favorable outcomes were achieved because skilled surgeons were able to directly verify the 3D position of the tumor in the operating room using SKIA-Breast before proceeding with the operation.

Although both ARL and USL are non-invasive techniques, USL has limitations, such as the requirement for painless, anxiety-free, and non-bleeding localization methods, along with the necessity for additional depth information and increased communication between the radiologist and the surgeon. Therefore, among the two non-invasive methods examined in our study, ARL offers the advantage of direct verification by the surgeon in the operating room, enhancing convenience and satisfaction for the operator. This was also corroborated by our user convenience survey, which indicated that ARL lessens the workload for radiologists by eliminating the need for procedures like carbon targeting, wire localization, and US marking before surgery.

As our study was exploratory, further studies investigating the clinical applications for AR-based localization are warranted. Most patients included in our study presented with a mass, but there is a need for studies that focus on non-mass presenting lesions that may not be visible on CT scans, as well as follow-up research on conditions like ductal carcinoma in situ and invasive lobular carcinoma. Although our study had a small sample size, it is anticipated that constraints such as selection bias can be mitigated in future studies by involving a larger patient cohort.

## Conclusion

BCS employing AR-guided localization demonstrated comparable results to those achieved using the traditional method of US-guided skin marking. We anticipate that surgeons who adopt the AR-based localization technique will find it an intuitive and convenient alternative to the established US skin marking method.

### Supplementary Information

Below is the link to the electronic supplementary material.

Supplementary file1 (mp4 60236 KB) Video: On the day of surgery, a screen recording from the iPad demonstrates the surgeon performing AR-based localization using the SKIA application. The entire process of applying AR-based localization in the operating room takes about 2 min

## Data Availability

The datasets generated during and analyzed during the current study are not publicly available due to concerns about exposing sensitive personal information. Still, they are available from the corresponding author upon reasonable request.

## Abbreviations

AR: Augmented reality
BCS: Breast-conserving surgery
CT: Computed tomography
MRI: Magnetic resonance imaging
OST: Optical see-through
ROLL: Radio-occult lesion localization
US: Ultrasound
VST: Video-see-through
3D: Three-dimensional
